# Cognitive biases in Mohs micrographic surgery

**DOI:** 10.1002/ski2.466

**Published:** 2024-12-01

**Authors:** Faisal Dubash, Andrew Affleck, Marese O’Reilly, Kenneth G. Gross

**Affiliations:** ^1^ Department of Dermatology Ninewells Hospital and Medical School Dundee UK; ^2^ Skin Surgery Medical Group Inc San Diego California USA

## Abstract

Mohs surgeons' fallibility is an important topic to explore. There are no studies on the role of cognitive biases in heuristic decision‐making within Mohs micrographic surgery (MMS) as a contributing factor in mistakes or errors of clinical judgement. We reviewed established cognitive biases applied to heuristics during the pre‐operative, intraoperative and reconstruction stages of MMS, and recommend conscious cognitive bias mitigating strategies for Mohs surgeons. Clinical errors/incidents may be reframed as opportunities to learn and develop, rather than evidence of competency or status. These experiences offer the opportunity to sharpen skills within metacognition and allow for more cognizant Mohs surgeons. We highly recommend all Mohs surgeons develop in‐depth knowledge and maintain awareness of cognitive biases throughout clinical practice, to enhance clinical performance.

**What is already known?**

- Mohs surgeons' fallibility is an important topic to explore. There are no studies on the role of cognitive biases in heuristic decision‐making within Mohs micrographic surgery (MMS) as a contributing factor in mistakes or errors of clinical judgement.

**What does this study add?**

- We reviewed established cognitive biases applied to heuristics during the pre‐operative, intraoperative and reconstruction stages of MMS, and recommend conscious cognitive bias mitigating strategies for Mohs surgeons.
- Clinical errors/incidents may be reframed as opportunities to learn and develop, rather than evidence of competency or status. These experiences offer the opportunity to sharpen skills within metacognition and allow for more cognizant Mohs surgeons.
- We highly recommend all Mohs surgeons develop in‐depth knowledge and maintain awareness of cognitive biases throughout clinical practice, to enhance clinical performance.

## 1 COGNITIVE BIASES IN MOHS MICROGRAPHIC SURGERY

Mohs micrographic surgery (MMS) was a major advancement in the surgical management of skin cancer. Over the years, the technique of MMS has been applied worldwide and its potential uses have expanded. Outcomes are generally excellent with cure rates equal to or higher than rates reported utilizing other treatment modalities but with further advantage of sparing of non‐involved tissues. MMS is a uniquely challenging experience. We are unaware of any other procedure where the clinician acts as the excisional surgeon, histopathologist and often the reconstructive surgeon. MMS is exacting and requires that the Mohs surgeon has procedural, cognitive and emotional competence for successful outcomes. MMS is both an art and science, and errors can arise leading to suboptimal outcomes. Previous discussion in the literature scoped possible causes of error, including poor surgical technique, problems with specimen processing and misinterpretation of histopathology slides.^1^, ^2^ We are unaware of any studies on the role of cognitive biases applied to heuristics in decision‐making within Mohs micrographic surgery (MMS) as a contributing factor in mistakes or errors of clinical judgement. We reviewed established cognitive biases^3^, ^4^, ^5^ applied to heuristics during the pre‐operative, intraoperative and reconstruction stages of MMS. We also recommend conscious cognitive bias mitigating strategies for Mohs surgeons.

Reflective practice is an excellent way to explore clinical processes and is an important part of clinical governance. When suboptimum outcomes arise, retrospective evaluation of clinical reasoning and decision‐making can unmask the contributing factors to errors which, broadly speaking, can be classified as due to cognitive biases or a consequence of knowledge deficits. In practice, there is often a combination/overlap that can be hard to untangle. Recurrent themes exist and conscious measures to attempt to identify possible unhelpful cognitive biases both prospectively and retrospectively are desirable. Each step of MMS involve decisions influenced by human factors (defined as environmental, organizational or occupational factors and human or individual characteristics), which impact performance and outcomes. An added level of complexity and stress may occur when multiple patients are having MMS at the same time and the Mohs surgeon transitions between rooms performing different tasks on different patients—surgical excisions, slide reading and reconstructions. If there are multiple challenging cases, increased stress may adversely affect task performance. We reviewed cognitive biases (which may be either conscious or subconscious) during three core stages of Mohs surgery (excision, slide interpretation and reconstruction).^6^

1. Excisional Mohs surgery

**Surgeon's choice of patients for MMS: High or Low Threshold, influenced by patient age**—unwilling to operate on the elderly due to a previous challenging experience when the patient struggled to tolerate MMS and became distressed and agitated (availability bias and representative bias).

**Type of skin cancer—**unwilling to perform MMS on squamous cell carcinoma (SCC) due to a previous case where there was a local recurrence, resulting in doubt as to the efficacy of MMS in such cases (availability bias).

**Site of Skin Cancer** and/or **Difficult Cases** such as large or aggressive tumours or tumours in difficult anatomical locations being referred to a colleague (underconfidence bias/cherry‐picking bias).

**Choice of Margins** (both deep and peripheral)—influenced by previous experience or the need to ‘get the tumour out at all costs’, taking an excessive margin to maximize chance of clearance (commission bias, availability bias). In contrast, omission bias is seen when the Mohs surgeon is nervous about the defect size and/or the complexity of reconstruction, leading to ill‐advised over‐preservation of the tissue.

**Debulking Issues**—debulking may make tissue handling and flattening easier but result in the disruption of the epidermal edge and deep margin (button‐holing artefact).

**Wrong Site Surgery**—should be avoided by using established systems including surgical pause and checklists, biopsy site photography and measurements taken from 3 skin landmarks to the biopsy site.^6^

2 Slide Interpretation

During slide interpretation several cognitive biases may occur. There is variation in how the threshold of diagnostic uncertainty is managed. The initial impression is formed by visual appraisal of low‐power scanning magnification mostly by pattern recognition. This is a fast, intuitive and almost unconscious way of thinking (Type 1 thinking). Unless a slower, conscious and effortful thinking process (Type 2) is also used in a reflective and analytical way, errors may arise if the initial Type 1 generated impression is incorrect. Conversely, false positives can arise if the slides are studied for too long and the Mohs surgeon starts to see things that are not there. Prolonged procrastination should prompt a second opinion. Errors due to cognitive biases are unlikely to be reduced by increased knowledge. Speeded tasks such as reading the slides very quickly or distractions will compromise Type 2 analytical study and can lead to errors. These errors may be corrected by learning to recognize cognitive biases and to actively invoke analytical strategies to correct any resultant errors. Conversely, if errors are a consequence of knowledge deficits, then more experience will result in fewer errors and speeded tasks will have minimal effect on accuracy, as seen in experienced Mohs surgeons and histopathologists who have an almost effortless way of interpreting the slides quickly and accurately. Errors due to lack of experience will be corrected by applying specific knowledge.^7^, ^8^

List of common cognitive biases that may cause error(s) in slide interpretation (Table 1).^3^, ^4^, ^5^

**TABLE 1:** Common cognitive biases in slide interpretation during Mohs surgery and their clinical significance.

Common cognitive biases in slide interpretation during Mohs surgery	Clinical significance
Availability bias	Recent experience influences management due to ease of recall and incorrectly perceived importance. Perceived bad outcomes are remembered more than good outcomes due to attached emotional element, for example, a recently missed superficial BCC prompts excessive stages to remove a focus of actinic keratosis.
Confirmation bias	Interpreting information to fit a preconceived diagnosis rather than the converse, for example, assuming a basaloid island is a BCC rather than considering other causes
Overconfidence bias	For example, a reluctance to a second opinion as a clinical quality check. This is the opposite of underconfidence bias when the Mohs surgeon asks too often for a second opinion for fear of error.
Search satisfying	Ceasing to look for further abnormalities when the first abnormality is found, for example, missing a separate subtle focus of tumour after detection of the main obvious tumour/‘calling off the search prematurely’.
Diagnostic momentum	Continuing to chase a tumour with multiple stages without challenging the diagnosis and changing the plan if required, for example, chasing a superficial BCC on the ear to the point where it compromises function instead of considering stopping the procedure and using topical imiquimod therapy.
Commission bias	Tendency towards action rather than inaction for example, taking an extra stage as it is ‘better to be safe than sorry’ despite apparent narrow clearance.
Omission bias	Tendency towards inaction rather than action, for example, interpreting the slides as just clear by 1 wafer/50 microns.
Outcome bias	A form of affective error—to prefer the outcome that is hoped for, for example, wrongly call a stage clear of cancer when everyone is tired and it is late in the day, when objectively it is not clear.
Representativeness bias	Not recognising an unusual BCC variant
Playing the odds	Risking a false negative interpretation by accepting a section with 80% peripheral margin visualized rather than 100%.

Histopathological pause—always having the biopsy slide at the Mohs microscope and compulsively confirm patient name, biopsy number and that the Mohs slides are in correct order before starting to read slides. There may be slight variations in approach to slide interpretation with currently no standardized reporting format in MMS with operation reports often documented in a narrative form, making them unintentionally error‐prone and susceptible to the omission of core data. Synoptic histology reporting is already used in reports for excised skin cancers, for example, melanoma and more recently, squamous cell and basal cell carcinomas. To ensure consistency, MMS reporting should incorporate synoptic histology reporting to achieve a minimum standard dataset comparable to existing pathology reports.^9^ From our Mohs experience, it is useful to read alternate wafers clockwise/counter clockwise and to start with the first wafer rather than the last. This helps mitigate pre‐emptive decisions and promotes a systematic approach with sequential slide analysis.

3 Reconstruction after Mohs Surgery

Mohs surgeons may exhibit multiple cognitive biases which influence reconstruction. Flapophilia—performing excessive number of flaps or complicated flaps when a simpler alternative would be better (overconfidence bias, ego bias and commission bias). Flapophobia (underconfidence bias and omission bias)—a hesitancy to perform skin flaps when they are indicated. Excessive use of KISS (Keep It Simple Stupid) leading to excessive use of secondary intention healing (SIH). Excessive need to always resurface defects and a very high threshold for SIH (ego bias and commission bias). Avoiding a specific local skin flap due to a poor outcome from one previous attempt (representative bias).

Outcome bias may result in decision‐making judgement based on the results of a previous procedure; such as a poor functional and cosmetic outcome of a previously performed local skin flap, rather than the quality of the process itself or the decision at the time. When the previous outcome was favourable, the decision maker's quality of thinking and competence is more likely to receive approval even though it may have been a risky decision influenced by good luck, and in reality, a different decision with less risk would have been preferable.^10^

The emotional state of the Mohs surgeon may influence clinical decision making in all 3 steps above and this may not be recognized. Sources of emotional influence on clinical performance include ambient‐induced factors, for example, environmental, interruptions, stress, lack of breaks, fatigue, and hunger. Endogenous factors also influence emotions with potential impact on clinical performance and include circadian, infradian and seasonal mood variation, mood disorders, anxiety disorders and burnout. Interactions with staff and patient can also result in unhelpful emotional influences including counter transference, fundamental attribution error (leading to an incorrect negative judgement of the behaviour of others based on their perceived disposition rather than situational circumstances) and specific emotional biases. This can result in persistent strained interpersonal relationship and interaction with associated stress for those involved. Stress, no matter the cause, can result in several physical symptoms including fatigue, blurred vision, eyestrain, difficulty focusing, headache, backache and muscle tension, which may contribute to loss of concentration that may result in error or poor judgement and performance in any Mohs tasks.^11^

Some cases of MMS need multiple stages to get clearance. It can be tempting to stick with the plan and continue until this is achieved but if it is very late and the patient and staff are tired, it may be better to pause, reflect, discuss and potentially postpone for another day or refer to another specialty if it is becoming technically very challenging or difficult to anaesthetize adequately. The Mohs surgeon could get ‘caught up in the moment’ becoming blinkered that they have to clear the tumour as they have invested so much time and energy on the case and not ‘stand back and admit defeat’, seeing that the desired result is not being achieved. This is known as sunk cost bias and is driven by the ego; not wanting to be wrong or seen as incompetent. There is overlap in this case scenario with egocentric bias when one relies too heavily on one's own perspective.

## 2 STRATEGIES FOR REDUCING ERRORS IN CLINICAL REASONING IN MMS^12^

1. Cognitive forcing strategies—to actively consider which cognitive biases may be active in the moment.
2. Slowing down and promote Type 2 thinking—this is generally helpful although it does not always improve interpretation and in some cases one can incorrectly start to doubt the initial Type 1 diagnosis.
3. Metacognition and considering alternatives—this raises awareness and insight into one's own thought processes. Specific questions are useful including, ‘What else could this be?’, ‘Is there anything that doesn't fit?’, ‘What are the reasons I think this is a BCC?’. ‘Is it possible that there is more than one abnormality?’, ‘If I am wrong what is the outcome going to be?’, ‘What is the worst case scenario here?’
4. Checklists—for reference—of slide quality, use of a minimum dataset for Mohs reporting, to compare and contrast two entities listing the discriminatory features, for example, BCC versus hair follicle, BCC versus vessels, BCC versus eccrine glands, BCC versus syringoma and DFSP versus scar tissue.
5. It is desirable to schedule regular Q&A sessions of Mohs cases with the consulting dermatopathologist and Mohs histotechnician.
6. Use of integrated validated artificial intelligence (AI) technologies to mitigate bias, for example, estimating tumour boundaries or assisting a Mohs surgeon with histology interpretation.^13^
7. Checking histopathological concordance with cold post‐Mohs reporting is a way to check accuracy and to look for any trends, for example, tending to over‐read features as tumour, therefore having false positive reports and unnecessary extra stages, or to under‐read and thus have false negative reporting/incomplete excisions. Cases of discordance can be studied and learning outcomes can be generated.
8. Group huddles during Mohs to check progress, asking all the team members (scrub nurse, health care assistant and biomedical scientist) for feedback on how the day is going, how they are all feeling, recognising any human factors contributing to the experience, and to agree on a plan. Emotional intelligence of all team members helps to facilitate the discussions.

## 3 CONCLUSION

Cognitive biases may contribute to errors and poor decisions during Mohs surgery. Such incidents may be reframed as opportunities to learn and develop, rather than evidence of competency or status. These experiences offer the opportunity to sharpen skills of metacognition and allow for more cognizant Mohs surgeons. We highly recommend all Mohs surgeons develop in‐depth knowledge and maintain awareness of cognitive biases throughout clinical practice, to enhance clinical performance.

## CONFLICT OF INTEREST STATEMENT

None declared.

## AUTHOR CONTRIBUTIONS

**Faisal Dubash**: Conceptualization (equal); formal analysis (equal); methodology (equal); project administration (equal); writing—original draft (equal); writing—review and editing (equal). **Andrew Affleck**: Conceptualization (equal); data curation (equal); formal analysis (equal); methodology (equal); project administration (equal); supervision (equal); visualization (equal); writing—original draft (equal); writing—review and editing (equal). **Marese O'Reilly**: Visualization (supporting); writing—review and editing (supporting). **Kenneth G. Gross**: Supervision (equal); visualization (equal); writing—review and editing (equal).

## ETHICS STATEMENT

Not applicable.

## PATIENT CONSENT

Not applicable.

## Data Availability

Data sharing not applicable to this article as no datasets were generated or analysed during the current study.
